# 3,3′-Bis(chloro­meth­yl)-4,4′-dieth­oxy-1,1′-biphen­yl

**DOI:** 10.1107/S1600536814004528

**Published:** 2014-03-05

**Authors:** Hager Trad, Mohamed Salah Belkhiria, Mustapha Majdoub

**Affiliations:** a University of Monastir, Faculté de Pharmacie, Avenue Avicenne, 5019 Monastir, Tunisia; b University of Monastir, Faculté des Sciences, Avenue de l’Environnement, 5019 Monastir, Tunisia

## Abstract

The asymmetric unit of the title compound, C_18_H_20_Cl_2_O_2_, consists of a half-mol­ecule, the other half being generated by an inversion center, located at the mid-point of the benzene–benzene bond. Except for the two Cl atoms, all other atoms of the compound are nearly coplanar, with the atomic displacements from the mol­ecular mean plane ranging from 0.0037 (19) to 0.071 (2) Å. The two Cl atoms are in *trans* positions and are displaced with respect to the mean plane by 1.687 (2) and −1.693 (3) Å. The crystal packing is governed by van der Waals inter­actions.

## Related literature   

For general background and synthesis, see: Trad *et al.* (2006 ▶); Hrichi *et al.* (2013 ▶). For related structures, see: Huang *et al.* (2011 ▶); Trad *et al.* (2012 ▶).
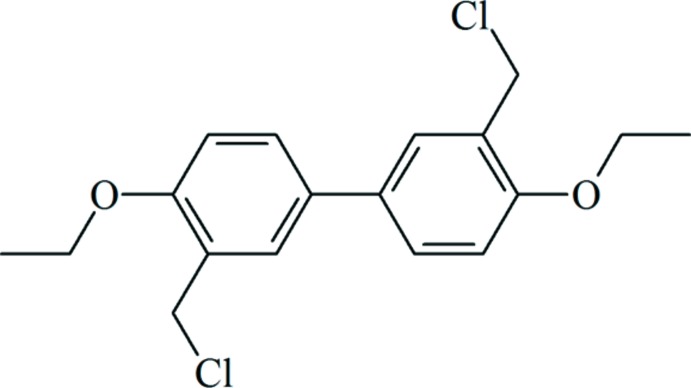


## Experimental   

### 

#### Crystal data  

C_18_H_20_Cl_2_O_2_
*M* *_r_* = 339.24
Monoclinic, 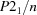

*a* = 4.984 (2) Å
*b* = 11.598 (5) Å
*c* = 14.578 (8) Å
β = 98.387 (2)°
*V* = 833.7 (7) Å^3^
*Z* = 2
Mo *K*α radiation
μ = 0.39 mm^−1^
*T* = 298 K
0.20 × 0.14 × 0.10 mm

#### Data collection  

Bruker–Nonius KappaCCD diffractometer
Absorption correction: multi-scan (*SORTAV*; Blessing, 1995 ▶) *T*_min_ = 0.937, *T*_max_ = 0.953
8808 measured reflections
2010 independent reflections
1252 reflections with *I* > 2σ(*I*)
*R*_int_ = 0.048

#### Refinement  

*R*[*F* ^2^ > 2σ(*F* ^2^)] = 0.048
*wR*(*F* ^2^) = 0.149
*S* = 1.05
2010 reflections
100 parameters
H-atom parameters constrained
Δρ_max_ = 0.26 e Å^−3^
Δρ_min_ = −0.28 e Å^−3^

### 

Data collection: *COLLECT* (Nonius, 2002 ▶); cell refinement: *DENZO*/*SCALEPACK* (Otwinowski & Minor, 1997 ▶); data reduction: *DENZO*/*SCALEPACK*; program(s) used to solve structure: *SIR2004* (Burla *et al.*, 2005 ▶); program(s) used to refine structure: *SHELXL97* (Sheldrick, 2008 ▶); molecular graphics: *ORTEP-3 for Windows* (Farrugia, 2012 ▶); software used to prepare material for publication: *SHELXL97*.

## Supplementary Material

Crystal structure: contains datablock(s) I, New_Global_Publ_Block. DOI: 10.1107/S1600536814004528/ds2239sup1.cif

Structure factors: contains datablock(s) I. DOI: 10.1107/S1600536814004528/ds2239Isup2.hkl

Supporting information file. DOI: 10.1107/S1600536814004528/ds2239Isup3.cml

CCDC reference: 988921

Additional supporting information:  crystallographic information; 3D view; checkCIF report

## Figures and Tables

**Fig. 1.**
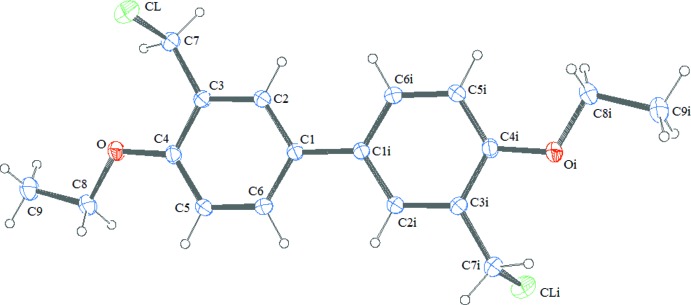
The molecular structure of the title compound BipEt2Cl2 with displacement ellipsoids drawn at 20% probability level for non hydrogen atoms.

**Fig. 2.**
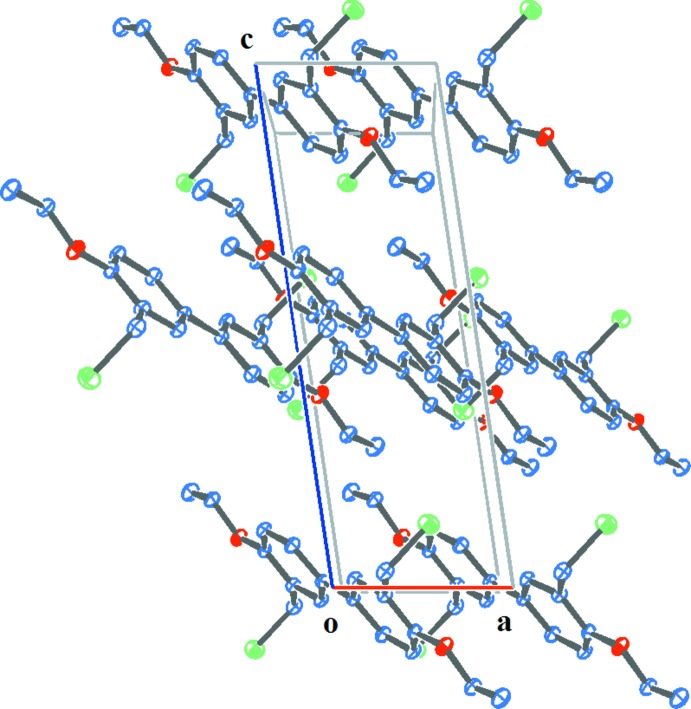
The crystal packing of compound BipEt2Cl2 viewed along b axis.

## supplementary crystallographic information

### 1. Comment

The synthesis of the compound 3,3'-Bis(chloromethyl)-4,4'-diethoxy-1,1'-biphenyl (BipEt2Cl2) is a part of an ongoing program on the investigation of new π-conjugated electroluminescent polymers derived from bisphenols (Trad *et al.*, 2006; Hrichi *et al.*, 2013).

The asymmetric unit of the title compound contains one-half of the molecule (Fig. 1) with the other half generated by an inversion center lieing between the two phenyl groups, at the the mid-point of the carbon-carbon bond. All atoms of the compound (BipEt2Cl2) lie in the same plane, the largest deviation being 0.0709 (22) Å for atom C9, except the two chlorine atoms. A π-conjugated system accounts for the planarity of the molecule and probably prevents the free rotation around the central carbon-carbon bond between the phenyl groups. The planes containing respectively the chloromethyl group and the biphenyl group are nearly orthogonal to each other, with a dihedral angle equal to 88.98 (9)°, such a value is nearly close to that observed for the 1-benzyloxy-2,5-bis(chloromethyl)-4-methoxybenzene (Trad *et al.*, 2012). The values of bond lengths and angles agree with those reported for similar compounds (Huang *et al.* 2011; Trad *et al.* 2012). A projection of the crystal structure of the compound, on the (010) plane, is given the by the figure 2.

### 2. Experimental

The compound 3,3'-Bis(chloromethyl)-4,4'-diethoxy-1,1'-biphenyl (BipEt2Cl2) was synthesized in two steps: a mixture of 4,4'-dihydroxy-1,1'-biphenyl (10 mmol), bromoethane (30 mmol) and anhydrous potassium carbonate (60 mmol) was added to 10 ml of DMF and was stirred for 24 h at room temperature. The reaction mixture was poured into distilled water and the intermediate product, 4,4'-diethoxy-1,1'-biphenyl (BipEt2), was extracted with diethyl ether, purified by recrystallization from ethanol/acetone (4/1) and then obtained as white fine powder. Yield: 80%; mp: 450–452 K. In a second step, a suspension of (BipEt2) (10 mmol) and paraformaldehyde (80 mmol), in a mixture of glacial acetic acid (30 ml) and 37% hydrochloric acid (8 ml), was left to stir for approximately 5 h at 328 K. After cooling, the resulting mixture was then poured into distilled water. The product (BipEt2Cl2) was extracted with diethylether and recrystallized from ethanol as colorless needle-like crystals. Yield: 65%; mp: 388 (2) K.

### 3. Refinement

All H atoms were refined using a riding model with C—H = 0.96 (CH3), 0.97 (CH2), 0.93 (CArH) Å and *U*_iso_(H) = 1.5 *U*_eq_(C), 1.2 *U*_eq_(C) and 1.2 *U*_eq_(C) respectively.

### Crystal data

**Table:**

C_18_H_20_Cl_2_O_2_	*F*(000) = 356
*M**_r_* = 339.24	*D*_x_ = 1.351 Mg m^−^^3^
Monoclinic, *P*2_1_/*n*	Mo *K*α radiation, λ = 0.71073 Å
Hall symbol: -P 2yn	Cell parameters from 8808 reflections
*a* = 4.984 (2) Å	θ = 2.3–29.0°
*b* = 11.598 (5) Å	µ = 0.39 mm^−^^1^
*c* = 14.578 (8) Å	*T* = 298 K
β = 98.387 (2)°	Needle, colourless
*V* = 833.7 (7) Å^3^	0.20 × 0.14 × 0.10 mm
*Z* = 2	

### Data collection

**Table:**

Bruker–Nonius KappaCCD diffractometer	2010 independent reflections
Radiation source: fine-focus sealed tube	1252 reflections with *I* > 2σ(*I*)
Graphite monochromator	*R*_int_ = 0.048
phi/w scans	θ_max_ = 28.1°, θ_min_ = 2.3°
Absorption correction: multi-scan (*SORTAV*; Blessing, 1995)	*h* = −6→6
*T*_min_ = 0.937, *T*_max_ = 0.953	*k* = 0→15
8808 measured reflections	*l* = 0→19

### Refinement

**Table:**

Refinement on *F*^2^	Primary atom site location: structure-invariant direct methods
Least-squares matrix: full	Secondary atom site location: difference Fourier map
*R*[*F*^2^ > 2σ(*F*^2^)] = 0.048	Hydrogen site location: inferred from neighbouring sites
*wR*(*F*^2^) = 0.149	H-atom parameters constrained
*S* = 1.05	*w* = 1/[σ^2^(*F*_o_^2^) + (0.077*P*)^2^ + 0.059*P*] where *P* = (*F*_o_^2^ + 2*F*_c_^2^)/3
2010 reflections	(Δ/σ)_max_ < 0.001
100 parameters	Δρ_max_ = 0.26 e Å^−^^3^
0 restraints	Δρ_min_ = −0.28 e Å^−^^3^

### Special details

**Table:**

Geometry. All s.u.'s (except the s.u. in the dihedral angle between two l.s. planes) are estimated using the full covariance matrix. The cell s.u.'s are taken into account individually in the estimation of s.u.'s in distances, angles and torsion angles; correlations between s.u.'s in cell parameters are only used when they are defined by crystal symmetry. An approximate (isotropic) treatment of cell s.u.'s is used for estimating s.u.'s involving l.s. planes.
Refinement. Refinement of *F*^2^ against ALL reflections. The weighted *R*-factor *wR* and goodness of fit *S* are based on *F*^2^, conventional *R*-factors *R* are based on *F*, with *F* set to zero for negative *F*^2^. The threshold expression of *F*^2^ > 2σ(*F*^2^) is used only for calculating *R*-factors(gt) *etc*. and is not relevant to the choice of reflections for refinement. *R*-factors based on *F*^2^ are statistically about twice as large as those based on *F*, and *R*- factors based on ALL data will be even larger.

### Fractional atomic coordinates and isotropic or equivalent isotropic displacement parameters (Å^2^)

**Table:**

	*x*	*y*	*z*	*U*_iso_*/*U*_eq_	
Cl	0.58814 (14)	0.13757 (6)	0.12470 (5)	0.0713 (3)	
O	0.5754 (3)	0.20148 (12)	−0.11362 (11)	0.0483 (4)	
C1	0.0870 (3)	0.45550 (16)	−0.01787 (13)	0.0345 (4)	
C2	0.1168 (4)	0.34506 (17)	0.01950 (15)	0.0391 (5)	
H2	0.0217	0.3264	0.0678	0.047*	
C3	0.2804 (4)	0.26160 (17)	−0.01136 (13)	0.0385 (5)	
C4	0.4206 (4)	0.28839 (17)	−0.08584 (14)	0.0388 (5)	
C5	0.3944 (4)	0.39671 (18)	−0.12463 (14)	0.0425 (5)	
H5	0.4869	0.4150	−0.1737	0.051*	
C6	0.2315 (4)	0.47848 (18)	−0.09126 (14)	0.0424 (5)	
H6	0.2173	0.5512	−0.1185	0.051*	
C7	0.3035 (4)	0.14484 (18)	0.03221 (16)	0.0493 (6)	
H7A	0.3263	0.0876	−0.0145	0.059*	
H7B	0.1377	0.1272	0.0568	0.059*	
C8	0.7054 (4)	0.2217 (2)	−0.19346 (16)	0.0500 (6)	
H8A	0.8346	0.2844	−0.1816	0.060*	
H8B	0.5720	0.2422	−0.2462	0.060*	
C9	0.8482 (5)	0.1126 (2)	−0.21296 (19)	0.0618 (7)	
H9A	0.9380	0.1234	−0.2663	0.093*	
H9B	0.7183	0.0513	−0.2248	0.093*	
H9C	0.9795	0.0932	−0.1603	0.093*	

### Atomic displacement parameters (Å^2^)

**Table:**

	*U*^11^	*U*^22^	*U*^33^	*U*^12^	*U*^13^	*U*^23^
Cl	0.0797 (5)	0.0631 (5)	0.0694 (5)	0.0113 (3)	0.0046 (3)	0.0182 (3)
O	0.0565 (9)	0.0371 (9)	0.0568 (9)	0.0086 (6)	0.0266 (7)	−0.0025 (7)
C1	0.0339 (10)	0.0325 (11)	0.0380 (10)	0.0000 (7)	0.0090 (7)	−0.0006 (8)
C2	0.0423 (10)	0.0346 (12)	0.0433 (11)	0.0010 (8)	0.0156 (8)	0.0015 (9)
C3	0.0444 (11)	0.0293 (11)	0.0435 (11)	−0.0001 (8)	0.0115 (9)	−0.0002 (9)
C4	0.0364 (10)	0.0359 (12)	0.0459 (12)	0.0019 (7)	0.0122 (8)	−0.0045 (9)
C5	0.0466 (12)	0.0398 (12)	0.0454 (12)	0.0020 (8)	0.0206 (9)	0.0042 (9)
C6	0.0485 (11)	0.0327 (11)	0.0490 (12)	0.0026 (8)	0.0169 (9)	0.0071 (9)
C7	0.0557 (13)	0.0360 (12)	0.0596 (15)	0.0039 (9)	0.0193 (11)	0.0031 (10)
C8	0.0524 (13)	0.0511 (14)	0.0503 (13)	0.0068 (10)	0.0206 (10)	−0.0039 (10)
C9	0.0685 (16)	0.0540 (16)	0.0679 (16)	0.0123 (11)	0.0267 (13)	−0.0088 (12)

### Geometric parameters (Å, º)

**Table:**

Cl—C7	1.811 (2)	C2—C3	1.382 (3)
O—C4	1.366 (2)	C3—C4	1.409 (3)
O—C8	1.432 (3)	C3—C7	1.493 (3)
C1—C2	1.391 (3)	C4—C5	1.376 (3)
C1—C6	1.399 (3)	C5—C6	1.382 (3)
C1—C1^i^	1.490 (3)	C8—C9	1.499 (3)
			
C4—O—C8	117.53 (16)	C3—C7—Cl	111.25 (15)
C2—C1—C6	115.93 (17)	C3—C7—H7A	109.4
C2—C1—C1^i^	122.3 (2)	Cl—C7—H7A	109.4
C6—C1—C1^i^	121.7 (2)	C3—C7—H7B	109.4
C3—C2—C1	123.47 (18)	Cl—C7—H7B	109.4
C3—C2—H2	118.3	H7A—C7—H7B	108.0
C1—C2—H2	118.3	O—C8—C9	107.42 (19)
C2—C3—C4	118.59 (18)	O—C8—H8A	110.2
C2—C3—C7	120.61 (18)	C9—C8—H8A	110.2
C4—C3—C7	120.78 (17)	O—C8—H8B	110.2
O—C4—C5	125.14 (18)	C9—C8—H8B	110.2
O—C4—C3	115.51 (17)	H8A—C8—H8B	108.5
C5—C4—C3	119.35 (17)	C8—C9—H9A	109.5
C4—C5—C6	120.47 (18)	C8—C9—H9B	109.5
C4—C5—H5	119.8	H9A—C9—H9B	109.5
C6—C5—H5	119.8	C8—C9—H9C	109.5
C5—C6—C1	122.17 (19)	H9A—C9—H9C	109.5
C5—C6—H6	118.9	H9B—C9—H9C	109.5
C1—C6—H6	118.9		

Symmetry code: (i) −*x*, −*y*+1, −*z*.
